# An Integrated Specialised Care Approach for Families with Multiple, Severe, and Enduring Problems: A Qualitative Evaluation

**DOI:** 10.5334/ijic.8576

**Published:** 2025-04-30

**Authors:** Anne Marie Barnhoorn-Bos, Eline Heek, Eva A. Mulder, Robert R. J. M. Vermeiren, Sarah Soenen, Inge Simons, Laura A. Nooteboom

**Affiliations:** 1 LUMC Curium –Department of Child and Adolescent Psychiatry, Leiden University Medical Center, Post Box 15, 2300 AA Leiden, The Netherlands; 2 GGZ Rivierduinen Children and Youth, Institute for Mental Health Care, Postbox 405, 2300 AK Leiden, The Netherlands; 3 Research Institute Child Development and Education, University of Amsterdam, Nieuwe Achtergracht 127, 1018 WS Amsterdam, The Netherlands; 4 Department of Child- and Adolescent Psychiatry and Psychosocial Care, AmsterdamUMC, Meibergdreef 5, 1105 AZ Amsterdam, The Netherlands; 5 Youz, Parnassia Group Psychiatric Institute, Dr. van Welylaan 2, 2566 ER, The Hague, The Netherlands; 6 De Banjaard, Outpatient Mental Healthcare Expertise Center for Youth with MID/BIF (Youz, Parnassia Group Psychiatric Institute), Dr. van Welylaan 2, 2566 ER, The Hague, The Netherlands; 7 Youz, Parnassia Group Psychiatric Institute, Dr. van Welylaan 2, 2566 ER, The Hague, The Netherlands

**Keywords:** integrated care, child and adolescent mental health, specialised care, families, professionals

## Abstract

**Introduction:**

To realize an integrated specialised care approach for families with multiple, severe, and enduring problems, specialised teams providing integrated care are emerging. In this qualitative evaluation, we aimed to gain insight into the key elements promoting an integrated specialised care approach at the level of professionals by studying five Specialist Integrated care Teams (SITs).

**Methods:**

Perspectives of families, professionals, managers, and policymakers were gathered by conducting 52 semi-structured interviews. Additionally, 40 observations of case meetings were conducted. A theory-driven framework method was applied to analyse the transcripts both deductively and inductively.

**Results:**

Five key elements promoting an integrated specialised care approach were identified: i) a broad view on families, ii) an integration of specialised perspectives, iii) providing specialised care flexible and timely, iv) working from families’ preferences and needs, and v) organising a well-structured care process and multidisciplinary evaluations.

**Discussion:**

When providing integrated specialised care, a family-centred focus, flexibility to tailor care within a well-structured care process, and investing in collaborative relationships with families are key.

**Conclusion:**

Although SITs are a promising form of an integrated specialised care approach, ensuring integrated care is the responsibility of every professional and (specialised) care service involved in families’ care.

## Introduction

Families with multiple, severe, and enduring problems across different life domains are in need for specialised support, but often fall between the cracks in a fragmented care system of siloed organisations [1]. This is not surprising, since the needs of these families on various life domains exceed the expertise of one organisation [22,3]. For example, specialised support for family members is often provided by separate youth or adult care services [2]. These separate care trajectories lead to fragmented care, as services lack the flexibility and joint coordination to collaboratively provide tailored care to families with complex needs [42,5]. Moreover, failing preconditions in terms of policy and finances hamper the provision of adequate support to this population in particular [52,6]. To improve integrated, specialised support for these families, there is a global trend towards the organisation of specific care initiatives, often in the form of fully integrated care teams [72,82,9]. In such teams, professionals from various disciplines and specialised organisations (e.g., youth and adult mental health care, intellectual disability care, and youth and parenting support) collaborate to provide outpatient support [10]. Although this seems a promising approach to overcome fragmentation and to provide specialised integrated care, little is known about this innovative practice. Hence, the aim of this study was to increase insight in a specialised integrated care approach, by studying five Specialist Integrated care Teams (SITs) that provide integrated care for families with complex needs.

Families with multiple, severe, and enduring problems comprise a heterogeneous group, facing varying and interacting problems on multiple life domains, including mental health problems, intellectual disabilities, financial problems, and parenting issues [1]. Consequently, each family presents its own unique combination of problems and protective factors of both children and parents, requiring a flexible and tailored approach to address a family’s specific needs [11]. Moreover, the co-occurrence and interactions between the problems of youth and parents demand a family-centred approach, which entails more than solely involving parents and other family members in a youth’s specialised treatment. It rather implies an additional focus on the interaction of problems of youth, parents, and other relatives to address all needs in an integrated context: a specialised integrated care approach [12].

Traditionally, specialised care services are organised separately, each with its own funding, legislation and practices. This hampers interprofessional collaboration between organisations and flexibility in care, that is needed to provide integrated support [132,14]. Moreover, it proves difficult for professionals to provide integrated care in practice, due to unfamiliarity with other professionals and their practices, barriers in communication and sharing information, and unclear roles and responsibilities [5].

To overcome fragmentation in care for families with multiple, severe, and enduring problems, specialised integrated care initiatives are emerging, ranging from multidisciplinary consultation and co-located care services to fully integrated care teams [102,132,142,15]. In this study, we focus on an emerging form of organising integrated support: Specialist Integrated care Teams (SITs). In these fully integrated care teams, professionals from various youth and adult specialised services (e.g. in the domains of mental health, youth and parenting support, intellectual disabilities, and addiction) collaborate on a daily basis to provide outpatient support to families with multiple, severe, and enduring problems (please find a more detailed description of the teams in section 2.1) [16].

As shown in previous research, providing integrated care is challenging, e.g. regarding allocating professional roles in care, sharing responsibilities, and addressing a broad spectrum of problems [5]. Moreover, the severity and unpredictability of family problems, which frequently leads to situations of crisis and insecurity, all too often puts additional pressure on interprofessional and inter-organisational collaboration [17]. Especially in complex cases, providing integrated care is impeded by emotional stress among professionals, disagreement on interventions to be deployed, and avoidance of tasks and responsibilities [182,19]. Hence, previous research showed that solely organising integrated care in multidisciplinary teams is insufficient to provide integrated care [20]. In fact, it requires adopting another working approach, e.g. in building shared thinking and mutual trust, developing a family-centred focus, and creating integrated care plans [5]. By following five SITs in the Netherlands in a qualitative evaluation study, our aim was to unravel key elements promoting an integrated specialised care approach at the level of professionals, by collecting perspectives of families, professionals, organisational managers, and policymakers. Moreover, to guide professionals in recognising which families might benefit from an integrated specialised care approach, we identified characteristics of families being supported by SITs based on descriptions and observations. With these insights, future initiatives may be guided in improving specialised integrated care for families with multiple, severe, and enduring problems.

## Method

### Setting and design

This study was part of the participatory action research project ‘The specialist nearby?!’ in which we followed five different SITs in the Netherlands over the course of two years (2020–2022) [3]. SITs provide integrated specialised care (i.e. diagnostics, treatment, and counselling) to families with multiple, severe, and enduring problems (e.g. mental health, parenting, social, and financial). SITs work locally to deliver specialised care close to families. The teams were all separate and locally organised initiatives from collaborating youth and adult care organisations, funded by local authorities and operating in various regions of the Netherlands (i.e. Haaglanden, Alphen aan de Rijn, Midden-Holland and Katwijk). The five teams showed differences regarding participating care organisations, funding arrangements, management and stage of development during the study (ranging from the initiative and design phase, the experimental and execution phase to the expansion and monitoring phase) (see Appendix A) [21]. Moreover, the composition of the teams varied in size (consisting of 3 to 16 professionals), and professionals’ occupation (comprising mostly child and parent social workers, and psychologists, but also systemic therapists, psychiatrists and youth physicians). Despite these organisational differences, all SITs shared a common aim for a similar target group, namely providing integrated specialised care to families with multiple, severe, and enduring problems. Moreover, the teams consist of comparable collaborating care organisations and expertise (i.e. all teams included professionals with expertise in youth and parenting care, youth mental health care and care for youth and adults with mild intellectual disabilities).

Due to the exploratory nature of this study and our aim to unravel key elements of an integrated specialised care approach in general, we analysed the five SITs together without distinguishing between different cohorts. We focused primarily on the shared elements of the SITs’ approaches promoting integrated specialised care for families with complex needs, as the small number of SITs in our study, with various developmental stages, organisations involved, financial constructions and compositions would not yield robust information to make meaningful statements on differences. To explore the complex reality of integrated care within a practice context we applied a qualitative design [22], comprising both semi-structured interviews with a wide range of participants and observations of SITs multidisciplinary case meetings [23]. In SITs multidisciplinary case meetings, care processes were evaluated with the various professionals involved, providing field information on the working approach of the SITs. By the triangulation of data (i.e. semi-structured interviews and observations), participants groups (i.e. parents, youth, professionals and policymakers/managers, from all SITs), and settings (five separate teams), the validity of our findings was fostered [24].

In this study, to engage the workforce in exploring and developing an integrated specialised care approach in practice, we involved three professionals from the SITs as research practitioners (as a side project during their daily task). This involved assisting in data collection by observing their own team’s multidisciplinary case meetings. Moreover, to connect the various teams in developing practice based knowledge and reflect on preliminary study results, we organised four learning sessions with professionals, policymakers, and managers of all SITs, as well as parents and youth representatives [252,26].

The Medical Ethics Review Board of Leiden University Medical Centre concluded that this research project was not subject to the Medical Research Involving Human Subject Act (WMO) and complied with the Netherlands Code of Conduct for Research Integrity (N20.200). The Consolidated criteria for Reporting Qualitative Research (COREQ) were applied to promote trans-parency and ensure clear and comprehensive reporting of the study methods [27].

### Participants

Participants, which included parents and youth, professionals, organisational managers, and local policymakers, were approached by the research practitioners of the SITs or directly by the researchers (EH and AB). To obtain a broad range of perspectives, we applied a purposive heterogeneous sampling method in which we predetermined the number of participants in each group and purposefully included participants with a wide variety of demographic characteristics and backgrounds [282,29]. Concerning families, we aimed for a representative mix of both parents and youth, with variation in gender, cultural background, family status, and educational level. Within the group of professionals, we included participants with varying expertise, occupation, educational level, work experience, and gender as much as possible. Policymakers from municipalities and managers from care organisations involved in the teams were included, with a mix of participants working close to the teams as well as participants with more distance from practice. From each SIT, an equal number of participants per group was recruited, parents and youth were counted as one group (i.e. families) (see Appendix C).

To avoid sampling bias, all families receiving care from the SITs were invited to participate in an interview by means of an accessible flyer, formulated in collaboration with a parent representative. To reach the often vulnerable and burdened families, recruitment of parents and youth was conducted by professionals from the teams. Clients were provided with verbal information and an additional letter describing the project and process of interviewing (audio-taping, confidentiality, and the right to withdraw at any time). The group of parents and youth willing to participate, largely matched the variation in demographics and background we aimed for. Professionals, policymakers, and managers were invited (by email, telephone or face-to-face) by the researchers (EH, AB) or the research practitioners of the SITs. After their written informed consent, participants were contacted to schedule the interview at their preferred location (at home, the team’s office or the researchers’ office) and modality (online or face-to-face). Necessary demographic data of all participants were documented before or during the interview, such as gender, age, cultural background, and educational level. Parents and youth received a gift voucher in acknowledgement of their participation.

### Data collection

Data were collected between March 2021 and April 2022 by semi-structured interviews and observations of multidisciplinary case meetings.

For the interviews, a topic list with open-ended questions (see appendix B) was formulated based on previous research [52,302,31] and exploratory interviews (N = 20) with practice experts on integrated specialised care. The topic list included questions on characteristics of the families in care and key elements of the teams’ support to families and was modified to fit the language and experiences of youth and parents, with help of a parent representative. In total, 52 interviews were conducted by two researchers (EH and AB) with parents (N *=* 18) and youth (N *=* 3), professionals (N *=* 20) organisational managers (N *=* 7), and local policymakers (N *=* 9). Four parent couples and a professional and organisational manager wished to be interviewed together, they were all counted as individual participants. Two parents requested to be interviewed in the presence of a professional for support; in order to include these parents’ stories, this was allowed as the professional remained in the background. The mean duration of the interviews was one hour. The interviews were audio-recorded and afterwards pseudonymised and transcribed (verbatim) by the researchers (AB and EH) and students (two of Medical University and one of University of Applied Sciences). The presented quotes have been translated from Dutch to English by the researcher (AB). Due to the verbatim transcription, the quotes presented in the results section contain literal wordings and therefore, might not be completely fluent.

Observations of multidisciplinary case meetings (N = 40) were monthly conducted by researchers (AB and EH) or by the research practitioners from the SITs. We applied an observation framework (see Appendix B) based on our literature review and exploratory interviews with practice experts on integrated specialised care. The observations focused on characteristics of families in care, aims, and key elements of the teams’ support to families. Each observation report was pseudonymised by the researchers.

To protect participants’ confidentiality, the audio recordings, transcripts and observation reports were archived in a data safe, accessible only to the project’s researchers. Documents (e.g., consent forms and demographic data) that could identify or link participants to the collected data were stored in a separate data safe.

### Analysis

The transcripts of the interviews were imported into ATLAS.ti (version 9), a qualitative data analysis software for labeling and organising textual data. A theory-driven framework method was applied to systematically code the transcripts both deductively and inductively [32]. The framework was based on a knowledge inventory entailing a (grey and academic) literature review and exploratory interviews with professionals, organisational managers, local policymakers and researchers (N = 20) on key elements of integrated specialised care for families with multiple, severe and enduring problems. We found key elements included (1) a broad view on family functioning, (2) collaborative relationships and shared decision-making with families, (3) continuity of care, and (4) involving families’ social network [12,52,302,31] (see Appendix B). After coding the transcripts on these predefined codes and family characteristics, open coding was applied to include all other potentially relevant themes. To enhance internal credibility of the results, all interviews were coded by two researchers (EH and AB) and any differences in codes were discussed and adjusted [242,27]. The observations of multidisciplinary case meetings were analysed through an observation framework (see Appendix B) and compared with the codes from the interviews for similar or new themes. In an iterative process throughout the data analysis, both the coding and observation framework were discussed and refined by the researchers (AB, EH, LN), adjusting code descriptions or adding new codes [322,33]. All interviews were coded, and after coding 25 interviews (an equal number of each participant group), no new themes emerged, indicating thematic saturation [32]. However, depth and nuances within the themes emerged when coding the remaining interviews. In analysing the data, we focused on the shared elements of an integrated specialised care approach from the perspectives of families, professionals, managers, and policymakers. We summarised and synthesised all analysed data (interviews and observations) by identifying the family characteristics and key elements and describing the patterns and themes within these key dimensions. A reflexive stance considering the researchers’ perspectives in generating and interpreting the data was fostered by keeping a research journal, noting reflections after each interview and observation, and by reflective meetings with the researchers (AB, LN, EH), the research practitioners, and a parent representative [34]. Informant feedback to increase internal credibility was obtained through learnings sessions on preliminary results with the participating professionals, managers, and policymakers as well as with parents and youth representatives [24]. These sessions also served as a member check [35].

## Results

This section starts with a short description of demographics of participants. Subsequently, we outline the main characteristics of the families being supported by SITs, based on the case meetings observations and interviews. Finally, we describe the SITs shared key elements promoting an integrated specialised care approach from the perspectives of parents and youth, professionals, policymakers, and managers.

### Demographics

Demographics of the youth, parents, professionals, managers, and policymakers can be found in Appendix C. The participating youth were all adolescent-aged yet varied in gender, educational level, and family structure. Parents varied in age (ranging from 30 to 59 years old), educational level, and family structure (e.g. two-parent and single parent households, raising one, two, or three and more children). Their cultural background was mainly Western and the majority of participating parents were identified female. The group of participating professionals showed variation in age (ranging from 30 to 65 years old), work experience (ranging from 0 to 49 years), educational level, expertise (e.g. youth mental health, youth and parenting support, intellectual disabilities care, and youth health service), and occupation (i.e. child and parent social workers, psychologists, systemic therapists, pediatric nurse, child psychiatrist, and youth physician). The majority of professionals were identified female. Both the policymakers and managers showed variation in age (ranging from 20 to 59 years old), work experience (ranging from 0 to 39 years), educational level, expertise (ranging from youth mental health, youth and parenting support, and youth health service), and occupation (diverse occupations); but not in gender (mainly identified female).

### Main characteristics of families supported by Specialist Integrated care Teams

Globally, five characteristics of the families in care of SITs were analysed. These characteristics are (1) problems among multiple family members and across different areas of life, which are (2) severe, enduring, and interrelated, leading to (3) frequent unsafety issues, (4) dysfunctional family dynamics, and a (5) long and troubled history of support. The characteristics are further outlined in Table 1.

**Table 1:** Main characteristics of families supported by SITs.


CHARACTERISTIC	DESCRIPTION

Problems among multiple family members and in different areas of life	Problems of youth and siblings in the family (e.g. in mental health, intellectual disability, addiction/crime, education/work), and Individual problems of the parents (e.g. in mental health, intellectual disability, addiction), and Socio-economic problems (e.g. finances, work, household, housing, social network).

Severe, enduring, and interrelated problems	Problems within the family are interwoven and mutually reinforcing. Problems fluctuate from periods of relative calmness to relapses and crises.

Frequent unsafety issues	High risk behaviour by the youth or the parent (e.g. aggression, suicidality, and addiction) Neglect, maltreatment or sexual abuse Unstable family situation due to socio-economic problems Youth welfare is regularly involved.

Dysfunctional family dynamics	Parental relational problems, parenting problems or adverse family dynamics Persistent intergenerational dysfunctional patterns

Long and troubled care history	Services fail to match family’s needs, leading to fragmented care. Distrust in care and lack of motivation Instability in coping capacity, with regard to daily life and managing the care process.


### Key elements of an integrated specialised care approach

To provide integrated care for the group of families with multiple, severe, and enduring problems, as described in Table 1, SITs adopt a specific working approach. We identified the SITs shared key elements promoting an integrated specialised care approach from the perspectives of families, professionals, managers, and policymakers. A summary of these five key elements is displayed in Table 2. In the following section, we describe each key element from the SITs practices and experiences, including facilitators and barriers in implementing the key element.

**Table 2:** Key elements of an integrated specialised care approach.


KEY ELEMENT	DESCRIPTION

Broad view on the family as a whole	Explore problems and strengths on various life domains, map out families’ care history and care network, and focus support on different family members.

Integration of specialist perspectives	Integrate different specialised expertises into an explanatory analysis of problems, together with families. Provide an integrated care plan combining specialised disciplines, aligned and prioritised to families’ needs.

Providing specialised care flexible and timely	Be flexible to families’ needs and preferences in intensity, timing, form, and variety of specialised care. Provide the required care timely.

Working from the preferences and needs of families	Adopt low-threshold approaches, connect to families’ life, strive for shared decision-making with all family members and focus on increasing self-efficacy and self-reliance.

Organising a well-structured care process, including continuous multidisciplinary and family evaluations	Structure the care process into different phases, monitor care in multidisciplinary meetings and family evaluations.


#### Broad view on the family as a whole

The first element of an integrated specialised care approach we identified was working from a broad view on the family as a whole, involving different specialist perspectives. This broad view was necessary to unravel and address the complexity of problems in family members and life domains. The SITs seemed to achieve this by explicitly taking time (in multiple meetings and over several weeks) to explore families’ problems, strengths, and protective factors on various life domains. In that, an important step was mapping out previous diagnostics and care trajectories, as families often had an extensive history in care. In addition, when formulating a care plan, support focused on multiple family members (i.e. the youth, parents, and siblings) and on dysfunctional dynamics within families. Furthermore, the social and care networks were identified and involved if necessary.

Often, youth are referred to care as identified problem. Therefore, professionals experienced that it takes time to change parents’ perspective form youth-centred to family-centred care and engage all family members in care. Moreover, youth described barriers to involve family members in care, as they felt ashamed about their own problems or guilty about the burden to be potentially causing on family members. Additionally, family conflicts were described as a barrier to involve the family as a whole.

“We are for the first time actually being seen by services as a family. So not as my daughter who has certain problems and a mother who has certain problems and being treated for it. But there is an integrated view on the family as a whole, as a two-unity.” Parent 2

#### Integration of specialised perspectives

A second key element emerging from the data was integrating specialised perspectives, which was considered essential to understand and address families’ multiple and interrelated problems. To achieve integration of specialised perspectives, professionals analysed families’ problems from various disciplines. In multidisciplinary case meetings, different perspectives were integrated into an explanatory analysis, which was then discussed with the family. Moreover, SITs provided integrated care plans with a combination of various specialised types of support, aligned and with a clear priority in order. The care plan could include youth and parent counselling (e.g. in parenting, coordination of care, finances, household, work), family therapy, parenting training, youth treatment (e.g. Cognitive Behavioural Therapy, vocational therapy or trauma therapy), parent treatment, and network strengthening interventions (e.g. Youth-Initiated Mentoring and Network Meetings).

A clear alignment and prioritisation in tasks, roles, and timing deemed necessary to avoid overwhelming the families with information and goals. Moreover, professionals emphasised to formulate explanatory analyses and integrated care plans with the family.

“The analysis phase is very important, in which different disciplines engage with all family members, bring it together in case meetings, explain it to the family, and consequently provide an interconnected care plan.” Professional 14

#### Providing specialised care flexible and timely

A third key element was the flexible provision of specialised care, which deemed essential to provide support timely and aligned to families’ specific needs.

SITs ensured flexibility in four different forms. First, they opted for flexibility in types of care: rather than applying standard protocols, families described interventions being adapted to their needs, capabilities, and coping capacities. Second, SITs were flexible regarding intensity of care, by scaling up and down frequency and type of interventions according to families’ needs at that time. Third, they provided flexibility in time, as the number and duration of appointments varied weekly, depending on families’ needs. Fourth, the SITs organised flexible collaboration, by involving professionals from other specialised disciplines when needed.

Professionals, policymakers, and managers mentioned professional autonomy as a precondition to provide flexible specialised care. This autonomy was facilitated by organisational preconditions, such as space and time to adapt to families’ needs in making appointments. Moreover, professionals were able to schedule appointments autonomously and were stimulated by both care organisations and policymakers to ‘colour outside the lines’. Professionals and families experienced SITs’ flexible and intensive engagement in families (e.g. in time and specialised disciplines) as a facilitator in quickly understanding dysfunctional family patterns and in building trust. However, the intensive provision of care could also prove a barrier, as it requires full commitment from families in time and effort. Although professionals considered their flexibility facilitating in addressing families’ needs, professionals as well as managers noted to be alert on professionals guarding their own boundaries in providing intensive and flexible care.

“By looking a step further than what you were used to do as a care professional, you hope to bring that little piece that families need.” Professional 11

#### Working from the preferences and needs of families

The fourth key element of an integrated specialised care approach we identified was working from the preferences and needs of families by a family-centred approach to build trust and engagement in care. This was considered important to align to families’ needs and address their often built-up distrust in care.

SITs had different ways in achieving a family-centred approach. First, they adopted a low-threshold approach by being easily approachable for families’ questions or acute problems. Second, SITs professionals connected to families’ own environment, e.g. by meeting at families’ home. Additionally, to broaden professional support into families’ everyday reality, the social network was actively involved. Third, SITs strived for shared decision-making on care with all family members. In shared decision-making, SITs aligned with families’ needs by taking families’ experiential knowledge seriously and supporting families in decision-making, e.g. by providing professional knowledge on options of care. Fourth, SITs focused on increasing families’ self-efficacy and self-reliance by enhancing the understanding of family members of their problems. Moreover, families’ coping capacities were increased by validating their strengths and highlighting even the smallest steps forward.

“If I want, we can also go to the office. But of course, it is nicer to have a coffee in a cafe and just talk. Then you feel more at ease as well.” Youth 3

#### Organising a well-structured care process, including continuous multidisciplinary and family evaluations

A final element was structuring the care process into different phases and monitoring the process in multidisciplinary case meetings. This enabled SITs to provide sufficient time and attention to the different key elements of an integrated specialised care approach.

The SITs structured the care process in four phases: the (1) admission phase, (2) analysis and familiarisation phase, (3) treatment and counselling phase, and (4) completion phase. In the admission phase, the SITs, referring professionals, and the family determined if SITs support was appropriate and necessary. The intensive analysis and familiarisation phase involved building trust and collaborative relationships, exploring families’ problems and needs, and creating an integrated care plan. During the treatment and counselling phase, the care plan was implemented by various professionals and, whenever necessary, adjusted in intensity or provided expertise. In the completion phase, SITs focused on empowering families and their network and organising follow-up care, if needed. Throughout the care process, multidisciplinary case meetings and families’ evaluations were planned to ensure integrating professional perspectives and aligning with families’ needs.

The duration of phases could be adapted to families’ needs, which allowed SITs to provide tailored support. However, due to contracts between care organisations and local municipalities, the duration of the overall care process was limited; in most teams at one year maximum. Sometimes insufficient time was available to achieve families’ goals, e.g. when significant time was required to build trust. A clear structure and direction in multidisciplinary case meetings was considered important to maintain overview and make joint decisions on care. Regarding families’ evaluations, professionals experienced barriers in scheduling regular evaluations, due to urgent family problems requiring attention or lack of priority by professionals.

“I think we really distinguish ourselves in trying not to get drawn into the crises, but to pause and take time to map out: Where is the family getting stuck, what has been tried before, and what is needed to break this pattern?” Professional 14

## Discussion

In this qualitative evaluation study of five Specialist Integrated care Teams (SITs) we found families supported by SITs, could be described by five characteristics: (i) families are experiencing problems among multiple family members and across different areas of life; (ii) which are severe, enduring, and interrelated; (iii) leading to frequent unsafety issues; (iv) dysfunctional family dynamics; and (v) a long and troubled care history. Moreover, we found that families, professionals, policymakers, and managers considered the following key elements essential in promoting an integrated specialised care approach, involving: (i) a broad view on the family as a whole, (ii) integrating specialised perspectives, (iii) providing specialised care flexible and timely, (iv) working from the preferences and needs of families, and (v) organising a well-structured care process.

Although the key elements are in line with previous research on integrated care for families [52,33], they provide a deeper understanding of what integrated specialised care implies for a specific group of families with multiple, severe, and enduring problems. First, a family-centred focus is necessary when providing integrated care. In these families, this is of specific importance since the problems are interwoven and need to be addressed in a combined treatment approach for both parents and youth [122,36]. Second, flexibility is essential to tailor care to families’ specific needs and wishes [11]. This is deemed important, due to the variety of problems and strengths that differentiate between each family and the fluctuation in severity of problems during a care process. Resonating the risk-need-responsivity model, risk and protective factors must be weighed for each family, to match the content and intensity of care to the family’s strengths and needs [37]. However, flexibility should be provided within a well-structured care process, guided by regular and effective multidisciplinary and family evaluations [52,38]. Finally, investing in collaborative relationships between family and professionals is key, by taking time in building trust and engaging families in care [392,40]. This was considered essential to overcome the distrust in care and lack of motivation often developed in these families after adverse previous experiences with care. Actively engaging families to ensure family-centred and tailored care, starts with gaining trust [41].

We explored an integrated specialised care approach in the specific setting of SITs. These teams seem promising in providing an integrated specialised care approach for families experiencing multiple, severe, and enduring problems. Because of the permanent, daily collaboration of professionals with different specialist expertise, integrating varying perspectives and providing integrated specialised care in a flexible and timely manner was facilitated. However, integrated initiatives in the form of a fully integrated team also have limitations. For example, to organize these initiatives, considerable investments (time and financial) are required to assemble the necessary care services, allocate appropriate resources, and define the population a team will focus on [42]. Moreover, SITs form a new, separate link in a family’s care process [9], as families are referred to SITs from other care services and the duration of SITs’ support is limited. This necessitates SITs to strongly invest in collaboration with other services in the care network, before, during, and after the SITs support. Ensuring continuity of care for families in this process will still be challenging [31], while SITs are intended to overcome fragmentation in care. Therefore, further research is necessary into the effectiveness of this form of care for families with multiple, severe, and enduring problems. Hence, more insight is needed on whether the form of fully integrated teams provides improved care, compared to e.g. collaboration between separate services or in collaborative networks [10]. By conducting mixed-method studies, combining quantitative and qualitative data, the effectiveness and implementation of integrated specialised care approaches should be further studied on different levels: the family, interprofessional, and inter-organisational collaboration [43].

Although SITs are a promising form of organising integrated care for families with complex needs, we believe an integrated specialised care approach is a responsibility for the whole care network that requires continuous reflection and adaptations at all levels (e.g. clinical, professional, organisational, policy) [102,44]. Specifically for professionals working with families with multiple, severe, and enduring problems, our findings can guide in recognising which families need this intensive and specialised approach to integrated care. In providing care to these families, professionals are encouraged to adopt the key elements of an integrated specialised care approach. This involves broadening their perspective to the whole family, investing in building trust and collaborative relationships with different family members, and applying flexible and low-threshold approaches in the care process. Moreover, professionals from different care services that collaborate within families, would benefit from arranging regular multidisciplinary meetings to integrate their specialised perspectives and working methods in integrated care plans.

Beyond the level of professionals, care organisations and policy play an important role in governing and facilitating an integrated specialised care approach [10]. For example, by providing professionals with the time and professional autonomy to build trustful relationships with families and flexibly respond to families’ needs. Moreover, multidisciplinary meetings to integrate professional perspectives must be facilitated in terms of organisation and funding to be embedded in families’ care process. To achieve appropriate care and policy, it is vital that families, professionals, organisations and policymakers collaboratively reflect, learn and adapt approaches where necessary. This requires long-term collaborations and investment in a learning system [62,44].

### Limitations and strengths of this study

A strength of this study is the qualitative design, in which we evaluated SITs as upcoming integrated initiatives from various perspectives (i.e. families, professionals, organisational managers, and local policymakers), based on multiple data sources (semi-structured interviews and observations), and from five separate initiatives in four different regions. This triangulation of data, participants groups and settings increased the validity of our findings [24].

Moreover, through our participatory design, we engaged research practitioners (professionals from SITs) and a parent representative as fellow researchers in designing the study, collecting data, and reflecting on results. This enabled us to gain deep insights in the complex practice of integrated care to highly-vulnerable families and develop practice-based knowledge [26]. However, there were also two teams who did not supply research practitioners, due to practical reasons (time or human resources) or their preference to have research conducted by external researchers. Because one designated researcher (EH) monthly observed the case observations and was in close contact with these two teams, we were still able to gain deep insight into these SITs’ daily practice. In addition, professionals in these teams appointed a contact person for the researchers (e.g., to approach families for participation) and provided one or more representatives participating in the learning sessions, that in other teams were tasks of the research practitioners. In this way, we have tried to overcome this limitation. Furthermore, the research practitioners were essential in approaching the burdened families to participate in interviews. In this way, we gathered a substantial group of participating parents. However, the number of participating youth is limited, which implies a limitation for this study. Although we encouraged research practitioners to invite all parents and youth, professionals felt inhibited by their fear of overburdening the family or doubts about parents’ or youths’ ability to participate. This may have affected the composition and size of the participant group.

The aim of our study, was to explore the SITs’ shared key elements of an integrated specialised care approach practice, as the teams largely shared the same goals and target group in their approach. However, the teams did differ in e.g. composition, organisation and developmental phase, which might influence their working approach. Therefore, we believe further research on differences between SITs and their impact on key elements of an integrated specialised care approach would provide highly relevant information on facilitators and barriers in implementing these elements.

### Conclusion

This study provides valuable knowledge for establishing an integrated specialised approach for families experiencing multiple, severe, and enduring problems. Although SITs are a promising form of organising this approach, ensuring integrated specialised care is the responsibility of every professional and specialised care service involved in families’ care. Families, professionals, organisations and policymakers should collaboratively reflect, learn and adjust approaches, to adopt the key elements of an integrated specialised care approach in practice.

## Additional File

The additional file for this article can be found as follows:

10.5334/ijic.8576.s1 Appendices. Appendix A to C.
