# Supplementary material for: An Integrated Specialised Care Approach for Families with Multiple, Severe, and Enduring Problems: A Qualitative Evaluation
Source: Int J Integr Care. 2025 Apr 30;25(2):6. doi: 10.5334/ijic.8576 (PMC12063601; doi:10.5334/ijic.8576)
Supplement: Appendices. — Appendix A to C. [file ijic-25-2-8576-s1.zip › ijic-8576_barnhoorn-bos-s1/Appendix+A_SITs+characteristics.docx]

**Appendix A**

**Table A.1**

*Target group, aim, organisation and composition of the SITs*

|  | Beter Thuis | In Verbinding | | PAST | | MAST | | Team in formation |
| --- | --- | --- | --- | --- | --- | --- | --- | --- |
| Region | Haaglanden | Midden-Holland | | Midden-Holland | | Alphen a/d Rijn | | Katwijk |
| Target group and eligibility criteria ^a^ | Families with multiple and  complex issues: mental health problems of youth (with possibly mild intellectual disabilities), combined with mental health problems or mild intellectual disabilities of parents, and difficulties on at least three domains of daily life (e.g. individual and family problems, parenting, social network, housing, finances, work/education). | Youth aged between 12 and 18 and their families with complex problems: involving (imminent) out-of-home placement, suspected unsafety and regular crisis situations, provided (regular) care has insufficient results.  The use of Youth Initiated Mentoring is appropriate. | | Families with complex problems, such as youth and parent mental health, parenting and daily life problems, with (imminent) unsafety, crisis or out-of-home placement, provided (regular) care has insufficient results. | | Youth aged 0-18 years with (suspected) complex mental health problems and/or mild intellectual disabilities and/or behavioral problems, complex problems and (suspected) mild intellectual disabilities of parents, parenting problems and family unsafety, disturbed daily life. | | Youth (aged 7+) with enduring (lifelong) and complex mental health problems, developmental disorders or mental disabilities and their families. |
| Aim ^b^ | Recovery of daily life through intensive family treatment | Establishing a support network around the family through informal support (Youth Initiated Mentoring) combined with integrated tailor-made treatment. | | Lifelong care through tailor-made provision of home care, counselling and treatment, with up- and downscaling when required. | | Preventing or minimising out-of-home placement through an intensive, systemic approach. | | Joint, well-organised and continuous care for children with complex problems. |
| Expertise of collaborating specialised care organisations | Youth mental health care  Care for youth and adults with mental disabilities  Youth - and parenting care  Adult mental health care  Adult counselling (daily life, work, finances)  Addiction care | Youth mental health care  Care for youth and adults with mental disabilities  Youth - and parenting care  Youth and adult counselling (daily life, work, finances) | | Youth and adult mental health care  Care and counselling for youth and adults with mental disabilities  Youth - and parenting care | | Youth mental health care  Care for youth and adults with mental disabilities  Youth - and parenting care  Youth and adult counselling (daily life, work, finances) | | Youth mental health care  Youth - and parenting care  General practitioner mental health care  Primary mental health care  Youth health care |
| Team composition and size ^c^ | 16 professionals (14 female, 2 male) | 6 professionals (6 female) | | 11 professionals (8 female, 3 male) | | 2 professionals (2 female) | | 8 professionals (7 female, 1 male) |
| Developmental stage of team ^d^ | Expansion and monitoring phase | Experimental and execution phase | | Experimental and execution phase | | Expansion and monitoring phase | | Initiative and design phase |
|  |  | |  | |  | |  |  |

*Note.*

^a^ Target group and eligibility criteria as described in SITs’ projectplans.

^b^ Aim as described by SITs.

^c^ Team composition varied through the period of data collection due to staff turnover, expansion of the team or care organisations leaving the integrated initiative. The reference date was taken at the end of the data collection.

^d^ Minkman et al., 2009
