# Supplementary material for: An Integrated Specialised Care Approach for Families with Multiple, Severe, and Enduring Problems: A Qualitative Evaluation
Source: Int J Integr Care. 2025 Apr 30;25(2):6. doi: 10.5334/ijic.8576 (PMC12063601; doi:10.5334/ijic.8576)
Supplement: Appendices. — Appendix A to C. [file ijic-25-2-8576-s1.zip › ijic-8576_barnhoorn-bos-s1/Appendix+B_+topic+lists+codetree+observation+framework.docx]

**Appendix B: Topic lists and code trees semi-structured interviews**

**Table B.1**

*Topic list semi-structured interview youth and parents*

| Topic | Question |
| --- | --- |
| Family characteristics | Gender  Age  Family status and composition  Educational level  Type of problems (global description)  Number of years of provided care to the family |
|  | How did you arrive to the SIT?  What care trajectory preceded this? |
| Aims and goals | What care do you receive from the SIT?  Does the provided care meet the needs of your family?  What are the most important goals of care for you?  What do you find important in the support you receive?  How do you and the professional ensure that the goals are achievable and match what is important to you and your family? |
| Experiences with the SIT | What is your experience so far with the SIT?  How do you view the provided care?  What makes this care different from the support you received before?  What do you think could improve the care provided by the team? |
|  |  |
| Broad view of family functioning | What life domains and which family members does the SITs care focus on?  Do you feel that the needs of all family members have been properly taken into account? |
| Collaborative relationships with the family | How do the team’s professionals manage to build collaborative relationships with the individual family members as well as with the family as a whole? |
| Continuity of care | Do you feel that the right help is available at the right time?  Do you feel that care can be provided flexible when needed? |
| Social network | How is your social network involved?  What are your wishes or needs in this? |

**Table B.2**

*Topic list semi-structured interview professionals, managers and policy makers*

| Topic |  | Question |
| --- | --- | --- |
| Demographics and expertise professionals | | Gender  Age  Educational level  Number of years of work experience  Organisation and expertise |
| Aims and goals | | What are the aims and focus of the SIT?  What should the SITs support to families with multiple, severe and enduring problems provide?  How does the SIT ensures that the goals of support are achievable and match what a family considers important? |
| Family characteristics | | For which families is the SIT intended?  And for which families not*?*  How can families addressed by the SIT can be characterised? |
| Experiences with the SIT | | What in the SITs working method works well or less well for this group of families?  What in the SITs working method do you consider an additional value to traditionally organised care? |
| Broad view of family functioning | | What life domains and which family members does the SITs care focus on?  How does the SIT take the needs of all family members into account? |
| Collaborative relationships with the family | | What is important in building collaborative relationships with these families?  How do you manage to build these relationships and do these families require different skills from you as a professional? |
| Shared decision-making | | How do you and the family make shared decisions, for example about the goals of support?  What do you think are important elements in shared decision-making with these families? |
| Continuity of care | | How is the continuity of care for the family guaranteed (during and after the SITs support)?  What’s the SITs role in care coordination? |
| Social network | | How is the social network of the family involved by the SIT? |

**Table B.3**

*Codes semi-structured interviews*

| Code | Description code |
| --- | --- |
| Aims of an integrated specialised care approach | Goals and focus of the SITs’ care to families with severe, multiple, and enduring problems  Feasibility of goals  Matching of goals with family’s needs and preferences |
| Characteristics of families being supported by an integrated specialised care approach | Characteristics of families being supported by SITs  Suitability criteria of families for SITs’ care |
| Key elements of an integrated specialised care approach | The SITs working approach in families; e.g. building collaborative relationships, shared decision-making, broad view on family functioning, continuity of care, and involving the social network  Working elements of an integrated care approach for families with severe, multiple and enduring problems  Additional value of an integrated specialised care approach |

| **Table B.4**  *Observation framework for multidisciplinary case meetings* | |
| --- | --- |
| SIT:  Date: |  |
| Characteristics of the case | Reason/question/aim of case discussion |
|  | Characteristics of the family in case: family composition, age of children, type of problems, requests for support, etc. |
|  | Support from the SIT being provided to the family  Disciplines and expertises provided from the SIT  Forms of support and frequency |
| Aims of care | Focus of support regarding family members and life domains |
|  | Aims and goals of support  Clarity of goals of care |
| Problem analysis/ explanatory analysis | Identified problems  Interrelatedness of problems  Agreement of professionals and family on the explanatory analysis of problems |
| Decision-making and prioritization | Process of setting goals and prioritising support  Family’s needs in decision-making |
| Monitoring/evaluation of support | Monitoring and evaluation of the care process by professionals and the family |
| Scaling up and down care in and outside the SIT | Reasons and plans for upscaling and downscaling care  Process of upscaling and downscaling care |
| Terminating the care process | Reasons for terminating the care process  Role and vision of professionals and family in terminating care |
| Role of professionals | Clarity and discussion about the role and commitment of each professional in a family’s care process |
| Preconditions for providing appropriate support to the family | In time, finances, availability of support |
| Exchange of information | Availability of information on the family from previous care  Exchange of information between professionals |
| Collaborative relationships with the family | Involvement of family members in the care process  Family’s control in the care process and in decision-making |
| Interprofessional collaboration in this case | Involvement of SITs professionals and expertises  Interprofessional collaboration around the family |
| Collaboration with the care network | Other care services involved in the family  Collaboration of the SIT with professionals and services in the care network outside the SIT  In referral to the SIT, during the SITs care process and at the termination of the SITs care or transfer to other services |
| Reflections or other observations | Other facilitators and barriers in the SITs working method with this family  Reflections on the case meeting |
