# Supplementary material for: An Integrated Specialised Care Approach for Families with Multiple, Severe, and Enduring Problems: A Qualitative Evaluation
Source: Int J Integr Care. 2025 Apr 30;25(2):6. doi: 10.5334/ijic.8576 (PMC12063601; doi:10.5334/ijic.8576)
Supplement: Appendices. — Appendix A to C. [file ijic-25-2-8576-s1.zip › ijic-8576_barnhoorn-bos-s1/Appendix+B_+topic+lists+codetree+observation+framework.pdf]

Appendix B: Topic lists and code trees semi-structured interviews

Table B.1

Topic list semi-structured interview youth and parents

Topic	Question
Family characteristics	Gender
	Age
	Family status and composition
	Educational level
	Type of problems (global description)
	Number of years of provided care to the family
	How did you arrive to the SIT?
	What care trajectory preceded this?
Aims and goals	What care do you receive from the SIT?
	Does the provided care meet the needs of your family?
	What are the most important goals of care for you?
	What do you find important in the support you receive?
	How do you and the professional ensure that the goals are achievable and match what is important to you and your family?
Experiences with the SIT	What is your experience so far with the SIT?
	How do you view the provided care?
	What makes this care different from the support you received before?
	What do you think could improve the care provided by the team?
Broad view of family functioning	What life domains and which family members does the SITs care focus on?
	Do you feel that the needs of all family members have been properly taken into account?
Collaborative relationships with the family	How do the team's professionals manage to build collaborative relationships with the individual family members as well as with the family as a whole?
Continuity of care	Do you feel that the right help is available at the right time?
	Do you feel that care can be provided flexible when needed?
Social network	How is your social network involved?
	What are your wishes or needs in this?

Table B.2

Topic list semi-structured interview professionals, managers and policy makers

Topic	Question
Demographics and expertise professionals	Gender
	Age
	Educational level
	Number of years of work experience
	Organisation and expertise
Aims and goals	What are the aims and focus of the SIT?
	What should the SITs support to families with multiple, severe and enduring problems provide?
	How does the SIT ensures that the goals of support are achievable and match what a family considers important?
Family characteristics	For which families is the SIT intended?
	And for which families not?
	How can families addressed by the SIT can be characterised?
Experiences with the SIT	What in the SITs working method works well or less well for this group of families?
	What in the SITs working method do you consider an additional value to traditionally organised care?
Broad view of family functioning	What life domains and which family members does the SITs care focus on?
	How does the SIT take the needs of all family members into account?
Collaborative relationships with the family	What is important in building collaborative relationships with these families?
	How do you manage to build these relationships and do these families require different skills from you as a professional?
Shared decision-making	How do you and the family make shared decisions, for example about the goals of support?
	What do you think are important elements in shared decision-making with these families?
Continuity of care	How is the continuity of care for the family guaranteed (during and after the SITs support)?
	What’s the SITs role in care coordination?
Social network	How is the social network of the family involved by the SIT?

Table B.3
Codes semi-structured interviews

Code	Description code
Aims of an integrated specialised care approach	Goals and focus of the SITs' care to families with severe, multiple, and enduring problems<br>Feasibility of goals<br>Matching of goals with family's needs and preferences
Characteristics of families being supported by an integrated specialised care approach	Characteristics of families being supported by SITs<br>Suitability criteria of families for SITs' care
Key elements of an integrated specialised care approach	The SITs working approach in families; e.g. building collaborative relationships, shared decision-making, broad view on family functioning, continuity of care, and involving the social network<br>Working elements of an integrated care approach for families with severe, multiple and enduring problems<br>Additional value of an integrated specialised care approach

Table B.4

Observation framework for multidisciplinary case meetings

SIT:	
Date:	
Characteristics of the case	Reason/question/aim of case discussion<br>Characteristics of the family in case: family composition, age of children, type of problems, requests for support, etc.<br>Support from the SIT being provided to the family<br>Disciplines and expertises provided from the SIT<br>Forms of support and frequency
Aims of care	Focus of support regarding family members and life domains<br>Aims and goals of support<br>Clarity of goals of care
Problem analysis/ explanatory analysis	Identified problems<br>Interrelatedness of problems<br>Agreement of professionals and family on the explanatory analysis of problems
Decision-making and prioritization	Process of setting goals and prioritising support<br>Family's needs in decision-making
Monitoring/evaluation of support	Monitoring and evaluation of the care process by professionals and the family
Scaling up and down care in and outside the SIT	Reasons and plans for upscaling and downscaling care<br>Process of upscaling and downscaling care
Terminating the care process	Reasons for terminating the care process<br>Role and vision of professionals and family in terminating care
Role of professionals	Clarity and discussion about the role and commitment of each professional in a family's care process
Preconditions for providing appropriate support to the family	In time, finances, availability of support
Exchange of information	Availability of information on the family from previous care<br>Exchange of information between professionals
Collaborative relationships with the family	Involvement of family members in the care process<br>Family's control in the care process and in decision-making
Interprofessional collaboration in this case	Involvement of SITs professionals and expertises<br>Interprofessional collaboration around the family

Collaboration with the care network	Other care services involved in the family<br>Collaboration of the SIT with professionals and services in the care network outside the SIT<br>In referral to the SIT, during the SITs care process and at the termination of the SITs care or transfer to other services
Reflections or other observations	Other facilitators and barriers in the SITs working method with this family<br>Reflections on the case meeting
