# Supplementary material for: An Integrated Specialised Care Approach for Families with Multiple, Severe, and Enduring Problems: A Qualitative Evaluation
Source: Int J Integr Care. 2025 Apr 30;25(2):6. doi: 10.5334/ijic.8576 (PMC12063601; doi:10.5334/ijic.8576)
Supplement: Appendices. — Appendix A to C. [file ijic-25-2-8576-s1.zip › ijic-8576_barnhoorn-bos-s1/Appendix+C_Demographics+of+participants.docx]

**Appendix C**

**Table C.1**

*Demographic characteristics of parents and youth*

| Parents (*n*=18) |  | Youth (*n*=3) |  |
| --- | --- | --- | --- |
| *Gender* |  | *Gender* |  |
| Male | 4 (22,2%) | Male | 2 (66,7%) |
| Female | 14 (77,8%) | Female | 1 (33,3%) |
| Non-binary | 0 (0%) | Non-binary | 0 (0%) |
| *Age* |  | *Age* |  |
| 30 – 39 years | 2 (11,1%) | 15 years | 1 (33,3%) |
| 40 – 49 years | 8 (44,5%) | 16 years | 1 (33,3%) |
| 50 – 59 years | 6 (33,3%) | 17 years | 1 (33,3%) |
| Unknown | 2 (11,1%) | Unknown | 0 (0%) |
| *Cultural background* |  | *Cultural background* |  |
| Western | 17 (94,5%) | Western | 2 (66,7%) |
| Non Western | 1 (5,5%) | Non Western | 1 (33,3%) |
| *Highest educational level* |  | *Highest educational level* |  |
| Secondary Vocational Education | 9 (50,1%) | High School | 1 (33,3%) |
| University of Applied Sciences | 6 (33,3%) | Secondary Vocational Education | 1 (33,3%) |
| University | 1 (5,5%) | University of Applied Sciences | 1 (33,3%) |
| Unknown | 2 (11,1%) | Unknown | 0 (0%) |
| *Family structure* |  | *Family structure* |  |
| Two-parent household | 12 (66,7%) | Two-parent household | 1 (33,3%) |
| Single-parent household | 6 (33,3%) | Single-parent household | 2 (66,7%) |
| *Number of children*  One child | 2 (11,1%) | *Number of children*  One child | 0 (0%) |
| Two children | 9 (50,1%) | Two children | 0 (0%) |
| Three or more children | 7 (38,8%) | Three or more children | 3 (100%) |
| *SIT/region ^a^*  Beter Thuis/Haaglanden  In Verbinding/Midden-Holland  PAST/Midden-Holland  MAST/Alphen a/d Rijn  Katwijk | 4 (22,2%)  4 (22,2%)  2 (11,1%)  5 (27,8%)  3 (16,7%) | *SIT/region*  Beter Thuis/Haaglanden  In Verbinding/Midden-Holland  PAST/Midden-Holland  MAST/Alphen a/d Rijn  Katwijk | 1 (33,3%)  1 (33,3%)  1 (33,3%)  0 (0%)  0 (0%) |

*Note.*

^a^ From each SIT, an equivalent number of participating parents and youth were recruited, parents and youth were counted as one group (i.e. families).

**Table C.2**

*Demographic characteristics of professionals, managers and local policymakers*

| Professionals (*n*=20) |  | Managers from care organizations (*n*=7) | | Policy makers from local municipalities (*n*=9) | |
| --- | --- | --- | --- | --- | --- |
| *Gender*  Male  Female  Non-binary |  | *Gender*  Male  Female  Non-binary |  | *Gender*  Male  Female  Non-binary | 1 (11,1%)  8 (88,9%)  0 (0%) |
|  | 1 (5%) |  | 2 (28,6%) |  |  |
|  | 19 (95%) |  | 5 (71,4%) |  |  |
|  | 0 (0%) |  | 0 (0%) |  |  |
| *Age*  20 – 29 years  30 – 39 years  40 – 49 years  50 – 59 years  60 – 69 years |  | *Age*  20 – 29 years  30 – 39 years  40 – 49 years  50 – 59 years  60-69 years |  | *Age*  20 – 29 years  30 – 39 years  40 – 49 years  50 – 59 years  60-69 years | 1 (11,1%)  4 (44,4%)  2 (22,2%)  2 (22,2%)  0 (0%) |
|  | 0 (0%)  8 (40%)  7 (35%)  4 (20%)  1 (5%) |  | 0 (0%)  2 (28,6%)  2 (28,6%)  3 (42,8%)  0 (0%) |  |  |
| *Work experience in years*  0 – 9 years  10 – 19 years  20 – 29 years  30 – 39 years  40 – 49 years |  | *Work experience in years*  0 – 9 years  10 – 19 years  20 – 29 years  30 – 39 years  40 – 49 years |  | *Work experience in years*  0 – 9 years  10 – 19 years  20 – 29 years  30 – 39 years  40 – 49 years | 3 (33,3%)  3 (33,3%)  3 (33,3%)  0 (0%)  0 (0%) |
|  | 5 (25%)  7 (35%)  5 (25%)  1 (5%)  2 (10%) |  | 3 (42,8%)  1 (14,3%)  1 (14,3%)  2 (28,6%)  0 (0%) |  |  |
| *Highest educational level*  Secondary Vocational Education  University of Applied Sciences  University |  | *Highest educational level*  Secondary Vocational Education  University of Applied Sciences  University |  | *Highest educational level*  Secondary Vocational Education  University of Applied Sciences  University | 0 (0%)  3 (33,3%)  6 (66,6%) |
|  | 1 (5%)  13 (65%)  6 (30%) |  | 0 (0%)  2 (28,6%)  5 (71,4%) |  |  |
| *Occupation*  Child and parent social worker  Psychologist/other therapist  Systemic therapist  Pediatric nurse  Child psychiatrist/youth physician |  | *Occupation*  Team/project manager  Director integrated care  Healthcare manager  Program manager (of region) | 2 (28,6%)  1 (14,3%)  2 (14,3%)  2 (28,6%) | *Occupation*  Municipal policy officer  Program manager  Contract Manager | 3 (33,3%)  4 (44,4%)  2 (22,2%) |
|  | 13 (65%)  4 (20%)  1 (5%)  1 (5%)  1 (5%) |  |  |  |  |
| *Expertise*  Youth mental health  Youth and parenting support  Intellectual disabilities  Youth health service | 4 (20%) | *Expertise*  Youth mental health | 3 (42,8%) |  |  |
|  | 9 (45%) | Youth and parenting support | 3 (42,8%) |  |  |
|  | 5 (25%) | Youth health service | 1 (14,3%) |  |  |
|  | 2 (10%) |  |  |  |  |
|  |  |  |  |  |  |
| *SIT/region*  Beter Thuis/Haaglanden  In Verbinding/Midden-Holland  PAST/Midden-Holland  MAST/Alphen a/d Rijn  Team in formation/Katwijk | 4 (20%)  3 (15%)  4 (20%)  4 (20%)  5 (25%) | *SIT/region*  Beter Thuis/Haaglanden  Midden-Holland *^a^*  MAST/Alphen a/d Rijn  Team in formation/Katwijk | 2 (28,6%)  3 (42,8%)  1 (14,3%)  1 (14,3%) | *SIT/region*  Beter Thuis/Haaglanden  Midden-Holland *^b^*  MAST/Alphen a/d Rijn  Team in formation/Katwijk | 3 (33,3%)  1 (11,1%)  3 (33,3%)  2 (22,2%) |

*Note.*

^a^ Managers of the SITs In Verbinding and PAST are counted as one group Midden-Holland, since they operated for both SITs.

^b^ Policy makers of the SITs In Verbinding and PAST are counted as one group Midden-Holland, since they operated for both SITs.
