# Supplementary material for: An Integrated Specialised Care Approach for Families with Multiple, Severe, and Enduring Problems: A Qualitative Evaluation
Source: Int J Integr Care. 2025 Apr 30;25(2):6. doi: 10.5334/ijic.8576 (PMC12063601; doi:10.5334/ijic.8576)
Supplement: Appendices. — Appendix A to C. [file ijic-25-2-8576-s1.zip › ijic-8576_barnhoorn-bos-s1/Appendix+C_Demographics+of+participants.pdf]

Appendix C

Table C.1

Demographic characteristics of parents and youth

Parents (*n*=18)		Youth (*n*=3)	
Gender		*Gender*	
Male	4 (22,2%)	Male	2 (66,7%)
Female	14 (77,8%)	Female	1 (33,3%)
Non-binary	0 (0%)	Non-binary	0 (0%)
Age		*Age*	
30 – 39 years	2 (11,1%)	15 years	1 (33,3%)
40 – 49 years	8 (44,5%)	16 years	1 (33,3%)
50 – 59 years	6 (33,3%)	17 years	1 (33,3%)
Unknown	2 (11,1%)	Unknown	0 (0%)
Cultural background		*Cultural background*	
Western	17 (94,5%)	Western	2 (66,7%)
Non Western	1 (5,5%)	Non Western	1 (33,3%)
Highest educational level		*Highest educational level*	
Secondary Vocational Education	9 (50,1%)	High School	1 (33,3%)
University of Applied Sciences	6 (33,3%)	Secondary Vocational Education	1 (33,3%)
University	1 (5,5%)	University of Applied Sciences	1 (33,3%)
Unknown	2 (11,1%)	Unknown	0 (0%)
Family structure		*Family structure*	
Two-parent household	12 (66,7%)	Two-parent household	1 (33,3%)
Single-parent household	6 (33,3%)	Single-parent household	2 (66,7%)

Number of children		*Number of children*	
One child	2 (11,1%)	One child	0 (0%)
Two children	9 (50,1%)	Two children	0 (0%)
Three or more children	7 (38,8%)	Three or more children	3 (100%)
SIT/region [a]		*SIT/region*	
Beter Thuis/Haaglanden	4 (22,2%)	Beter Thuis/Haaglanden	1 (33,3%)
In Verbinding/Midden-Holland	4 (22,2%)	In Verbinding/Midden-Holland	1 (33,3%)
PAST/Midden-Holland	2 (11,1%)	PAST/Midden-Holland	1 (33,3%)
MAST/Alphen a/d Rijn	5 (27,8%)	MAST/Alphen a/d Rijn	0 (0%)
Katwijk	3 (16,7%)	Katwijk	0 (0%)

Note.
[a] From each SIT, an equivalent number of participating parents and youth were recruited, parents and youth were counted as one group (i.e. families).

Table C.2

Demographic characteristics of professionals, managers and local policymakers

Professionals (*n*=20)		Managers from care organizations (*n*=7)		Policy makers from local municipalities (*n*=9)	
Gender		*Gender*		*Gender*	
Male	1 (5%)	Male	2 (28,6%)	Male	1 (11,1%)
Female	19 (95%)	Female	5 (71,4%)	Female	8 (88,9%)
Non-binary	0 (0%)	Non-binary	0 (0%)	Non-binary	0 (0%)
Age		*Age*		*Age*	
20 – 29 years	0 (0%)	20 – 29 years	0 (0%)	20 – 29 years	1 (11,1%)
30 – 39 years	8 (40%)	30 – 39 years	2 (28,6%)	30 – 39 years	4 (44,4%)
40 – 49 years	7 (35%)	40 – 49 years	2 (28,6%)	40 – 49 years	2 (22,2%)
50 – 59 years	4 (20%)	50 – 59 years	3 (42,8%)	50 – 59 years	2 (22,2%)
60 – 69 years	1 (5%)	60-69 years	0 (0%)	60-69 years	0 (0%)
Work experience in years		*Work experience in years*		*Work experience in years*	
0 – 9 years	5 (25%)	0 – 9 years	3 (42,8%)	0 – 9 years	3 (33,3%)
10 – 19 years	7 (35%)	10 – 19 years	1 (14,3%)	10 – 19 years	3 (33,3%)
20 – 29 years	5 (25%)	20 – 29 years	1 (14,3%)	20 – 29 years	3 (33,3%)
30 – 39 years	1 (5%)	30 – 39 years	2 (28,6%)	30 – 39 years	0 (0%)
40 – 49 years	2 (10%)	40 – 49 years	0 (0%)	40 – 49 years	0 (0%)
Highest educational level		*Highest educational level*		*Highest educational level*	
Secondary Vocational Education	1 (5%)	Secondary Vocational Education	0 (0%)	Secondary Vocational Education	0 (0%)
University of Applied Sciences	13 (65%)	University of Applied Sciences	2 (28,6%)	University of Applied Sciences	3 (33,3%)
University	6 (30%)	University	5 (71,4%)	University	6 (66,6%)
Occupation		*Occupation*		*Occupation*	
Child and parent social worker	13 (65%)	Team/project manager	2 (28,6%)	Municipal policy officer	3 (33,3%)
Psychologist/other therapist	4 (20%)	Director integrated care	1 (14,3%)	Program manager	4 (44,4%)
Systemic therapist	1 (5%)	Healthcare manager	2 (14,3%)	Contract Manager	2 (22,2%)

Pediatric nurse	1 (5%)	Program manager (of region)	2 (28,6%)		
Child psychiatrist/youth physician	1 (5%)				
Expertise		*Expertise*			
Youth mental health	4 (20%)	Youth mental health	3 (42,8%)		
Youth and parenting support	9 (45%)	Youth and parenting support	3 (42,8%)		
Intellectual disabilities	5 (25%)	Youth health service	1 (14,3%)		
Youth health service	2 (10%)				
SIT/region		*SIT/region*		*SIT/region*	
Beter Thuis/Haaglanden	4 (20%)	Beter Thuis/Haaglanden	2 (28,6%)	Beter Thuis/Haaglanden	3 (33,3%)
In Verbinding/Midden-Holland	3 (15%)	Midden-Holland [a]	3 (42,8%)	Midden-Holland [b]	1 (11,1%)
PAST/Midden-Holland	4 (20%)	MAST/Alphen a/d Rijn	1 (14,3%)	MAST/Alphen a/d Rijn	3 (33,3%)
MAST/Alphen a/d Rijn	4 (20%)	Team in formation/Katwijk	1 (14,3%)	Team in formation/Katwijk	2 (22,2%)
Team in formation/Katwijk	5 (25%)				

Note.

[a] Managers of the SITs In Verbinding and PAST are counted as one group Midden-Holland, since they operated for both SITs.

[b] Policy makers of the SITs In Verbinding and PAST are counted as one group Midden-Holland, since they operated for both SITs.
